# AAV-Mediated Overexpression of Neuroserpin in the Hippocampus Decreases PSD-95 Expression but Does Not Affect Hippocampal-Dependent Learning and Memory

**DOI:** 10.1371/journal.pone.0091050

**Published:** 2014-03-07

**Authors:** Vicky W. K. Tsang, Deborah Young, Matthew J. During, Nigel P. Birch

**Affiliations:** 1 School of Biological Sciences, University of Auckland, Auckland, New Zealand; 2 Department of Molecular Medicine & Pathology, University of Auckland, Auckland, New Zealand; 3 Department of Pharmacology and Clinical Pharmacology, University of Auckland, Auckland, New Zealand; 4 Centre for Brain Research, University of Auckland, Auckland, New Zealand; 5 Department of Molecular Virology, Immunology and Medical Genetics, Ohio State University, Columbus, Ohio, United States of America; Universidade do Estado do Rio de Janeiro, Brazil

## Abstract

Neuroserpin is a serine protease inhibitor, or serpin, that is expressed in the nervous system and inhibits the protease tissue plasminogen activator (tPA). Neuroserpin has been suggested to play a role in learning and memory but direct evidence for such a role is lacking. Here we have used an adeno-associated virus (AAV) vector expression system to investigate the effect of neuroserpin on hippocampal-dependent learning and memory in the young adult rat. A FLAG-tagged neuroserpin construct was initially characterized by *in vitro* transcription/translation and transfection into HEK293 cells and shown to interact with tPA and be targeted to the secretory pathway. Targeted injection of a chimeric AAV1/2 vector expressing FLAG-neuroserpin resulted in localized overexpression in the dorsal hippocampus. Neuroserpin overexpression led to the appearance of an unstable neuroserpin:tPA complex in zymographic assays consistent with interaction with endogenous tPA *in vivo*. Rats overexpressing neuroserpin also showed a significant decrease in the levels of postsynaptic density protein 95, a major postsynaptic scaffolding protein. Three weeks after injection, a range of behavioural tests was performed to measure spatial and associative learning and memory, as well as innate and acquired fear. These tests provided no evidence of a role for neuroserpin in hippocampal-dependent learning and memory. In summary this study does not support a role for neuroserpin in hippocampal-dependent learning and memory in young adult rats but does suggest an involvement of neuroserpin in hippocampal synaptic plasticity.

## Introduction

Neuroserpin was discovered as an axonally-secreted protein in chicken dorsal root ganglion cells and subsequently shown to be a member of the serpin superfamily of serine protease inhibitors [1]. Neuroserpin transcripts and protein are largely restricted to the nervous system, and expressed in neurons both during development and in the adult [1]–[3]. During embryonic development neuroserpin transcripts are expressed in most CNS regions, increasing to a maximal level perinatally before declining to a moderate level in the adult [3]. In the adult, neuroserpin is most strongly expressed in the hippocampus, neocortex, the olfactory bulb and the amygdala. The expression of neuroserpin in these regions which are known to display a high degree of plasticity has led to speculation that neuroserpin may play a role in the modulation of synaptic efficacy or structural changes associated with learning and memory [3]. Such changes could be through inhibition of its target protease tissue plasminogen activator (tPA) [2], [4]. The expression of neuroserpin in the nervous system coincides to a significant extent with tPA [2], [3], [5]. tPA has been shown to be associated with the maintenance of long lasting LTP in the hippocampus [6]–[10] and expression of plasminogen activators has been associated with performance in a range of learning tasks [7], [11]. Neuroserpin expression is increased in the developing visual cortex during the critical period of experience-dependent refinement of neuronal synaptic connectivity and decreased following monocular deprivation [12]. tPA activity is increased under the same conditions [13] raising the possibility that the balance between neuroserpin and tPA levels plays a role in the remodelling of these synaptic connections.

Further insights into a function for neuroserpin in synaptic plasticity have come from studies of transgenic mice overexpressing neuroserpin and neuroserpin knockout mice [14] which have implicated neuroserpin in the regulation of emotional behaviour. Neuroserpin-deficient mice show a selective reduction in motor activity in novel environments, an anxiety-like response on the O-maze and a neophobic response to novel objects. Mice overexpressing neuroserpin under the control of the Thy1.2 promoter show reduced centre exploration in the open field test and, similar to the neuroserpin-deficient mice, a neophobic phenotype in the novel object test. While the mechanism leading to the behavioural changes was not determined, the lack of any change in tPA activity levels in neuroserpin-deficient mice led the researchers to suggest that it is at least in part independent of tPA activity.

In this study we have used well established recombinant adeno-associated virus (AAV)-mediated gene transfer techniques [15], [16] to investigate a role for neuroserpin in another domain of behaviour, hippocampal-dependent learning and memory in young adult rats. We have injected a chimeric AAV1/2 vector combining the tropism of AAV serotype 1 and 2 [17], [18], to achieve localized long-term overexpression of neuroserpin in neurons in the dorsal hippocampus [19]. The effect of neuroserpin overexpression on tPA, PAI-1 and neuronal proteins linked to synaptic plasticity was investigated. Three weeks after injection, a range of behavioural tests was performed to measure locomotor activity, spatial and associative learning and memory, as well as innate and acquired fear.

## Materials and Methods

### Antibodies

The following antibodies were used in this study: Affinity-purified neuroserpin apK53, a rabbit polyclonal antibody raised to a synthetic peptide corresponding to the 19 C-terminal amino acids of rat neuroserpin [20], anti-FLAG (Cat No: F3165, Sigma-Aldrich Co. LLC, MO, USA), anti-GAPDH (Cat No: ab8245, Abcam, Cambridge, UK), anti-synapsin (Cat No: 611392, BD Biosciences, CA, USA), anti-PSD-95 (Cat No: MAI-045, Thermo Scientific, IL, USA), anti-NR1 (Cat No: 556308, BD Biosciences, CA, USA), anti-Tau (Cat No: MAB3420, Millipore, MA, USA), anti-GAP-43 (Cat No: G9264, Sigma-Aldrich Co. LLC, MO, USA), goat anti-rabbit IgG (H+L) Horseradish Peroxidase-conjugated antibody (Cat No: 111-035-003, Jackson ImmunoResearch Laboratories, Inc., PA, USA), and goat anti-mouse IgG (H+L) Horseradish Peroxidase-conjugated (Cat No: 115-035-003, Jackson ImmunoResearch Laboratories, Inc., PA, USA).

### Preparation of FLAG-tagged Neuroserpin Expression Constructs and AAV Vector Production

A FLAG epitope tag with an additional two alanine residues at the amino-terminal end was inserted into a rat neuroserpin cDNA (GenBank accession number: AF193015.1) after the signal sequence (i.e. after Ala_20_) by sequential PCR. The amplified sequence was adenylated at its 3′ ends and subcloned via the pGEM-T Easy vector into pcDNA3.1. The coding sequence was then subcloned into an AAV expression plasmid under control of a chicken-beta actin/CMV hybrid (CBA) promoter and containing a woodchuck post-transcriptional regulatory element (WPRE) and bovine growth hormone polyadenylation sequence (bGHpA) flanked by AAV2 inverted terminal repeats to generate pAAV-CBA-FLAG-NS-WPRE-bGHpA. The coding sequence in both vectors was confirmed by DNA sequencing. A humanized *Renilla reniforms* GFP (hrGFP) cDNA was cloned into the same pAAV backbone. Chimeric AAV1/2 viral vectors with virions containing a 1∶1 ratio of AAV1 and AAV2 capsid proteins [21] were generated as described previously [22]. In brief, HEK293 cells were transfected with the AAV plasmid (either the pAAV-CBA-polylinker-WPRE-bGHpA (empty vector), pAAV-CBA-hrGFP-WPRE-bGHpA or pAAV-CBA-FLAG-NS-WPRE-bGHpA plasmid), the AAV1 (pH21) and AAV2 (pRV1) helper plasmids, and the adenovirus helper plasmid (pFΔ6) using calcium phosphate. Cells were harvested and the vector purified using HiTrap heparin affinity columns (Sigma, St Louis, MO) sixty hours after transfection. Genomic titres (genome-containing viral particles) were determined by quantitative real-time reverse-transcriptase polymerase chain reaction (qRT-PCR) using the ABI 7700 real time PCR cycler (Applied Biosystems, Foster City, CA) with primers designed to WPRE.

### Expression of FLAG-tagged Neuroserpin by in vitro Transcription and Translation and by Transduction of HEK293 Cells

^35^S-radiolabelled neuroserpins, cloned into the pcDNA3.1 vector, were generated using the TNT T7 coupled reticulocyte lysate systems (Promega, WI, USA) according to the manufacturer’s instructions. Products were incubated with tPA at 30°C for 30 min in complexation buffer (133 mM NaCl, 67 mM Tris-HCl, pH 7.5), separated by SDS-PAGE and analyzed by autoradiography. The radioactive proteins were imaged by exposing the gels to BAS-IP MP imaging plates (Fuji Film Co. Ltd, Japan). HEK293 cells were maintained in Minimum Essential Medium (MEM, Invitrogen, CA, USA) with non essential amino acids, sodium pyruvate (110 mg/l), 10% fetal calf serum, 4 mM glutamine, 100 U/ml penicillin and 100 µg/ml streptomycin. Cells were plated in a 24-well plate at a density of 5×10^4^ cell/well. AAV viral particles (5×10^8^ particles) were added to the cells at a viral particle: cell ratio of 10,000∶1 twenty four hours later. Cell lysates were prepared 48 h after transduction. RIPA buffer (50 mM Tris-HCl pH 8.0, 150 mM NaCl, 1% NP-40, 0.5% sodium deoxycholate, 0.1% SDS, 2 mM EDTA) containing protease inhibitors (cOmplete Mini protease inhibitor cocktail, Roche New Zealand) was added to cells and incubated with gentle shaking for 15–30 min at 4°C. Lysed cells were then triturated before transferring to sterile microfuge tubes and centrifugation at 14,000×*g* for 30 min at 4°C. Protein concentrations in the supernatants were determined using the Bio-Rad DC Protein Assay (Bio-Rad Laboratories, CA, USA). Proteins (20 µg) were analysed by SDS-PAGE and western blotting as described previously [23] using affinity-purified neuroserpin apK53.

### Injection of AAV Vectors into the Rat Hippocampus and Analysis of Protein Expression

All animal experiments were approved by the University of Auckland Animal Ethics Committee (AEC approval: AEC/12/2003/R208). Male Wistar rats (250–300 g) were housed at 22°C with a 12 h light/dark cycle (lights out at 4 pm), with 2–3 animals per cage and food and water available *ad libitum*. Viral injection was carried out as previously described [24] with minor changes. Animals were anaesthetized with sodium pentobarbital (Pentobarb, Provet NZ Pty Ltd) followed by injection of Marcaine (Sigma-Aldrich Co. LLC, MO, USA) on the scalp as a local anaesthetic. Each vector (2 µl; AAV-empty = 5.0×10^11^ viral genomes/ml, AAV-hrGFP = 3.4×10^11^ viral genomes/ml, AAV-FLAG-NS = 6.7×10^11^ viral genomes/ml), mixed with 1 µl of 20% mannitol to enhance vector spread [25], was injected unilaterally or bilaterally into the dorsal hippocampus using the following coordinates from bregma (mm): anterior-posterior −3.8, medial-lateral ±2.0 and dorsal-ventral −4.1 from the surface of the skull [26] at a rate of 100 nl/min over 30 min (Figure S1A). Immunohistochemistry (IHC) was performed on free-floating brain sections, collected 21 days after AAV injection, as previously described [24]. For Western blot analysis, hippocampi from rats that had been subjected to the open-field test, the novel object recognition test and the Morris water maze were dissected 61 days after AAV injection. The hippocampi were snap frozen on dry ice and stored at −80°C until extraction. Lysate samples were prepared by homogenization in lysis buffer (50 mM Tris-HCl, 2 mM EDTA, pH 7.5, 1x cOmplete mini protease inhibitor cocktail mix (Roche, New Zealand); 10 µl per mg of hippocampus sample) using an Eppendorf Micropestle (Eppendorf, Hamburg, Germany). Lysed samples were centrifuged at 20,000×g for 30 min at 4°C and supernatants analysed by western blotting as described previously [23]. Deglycosylation was carried out using N-Glycosidase F (Roche, New Zealand). Protein samples (20 µg) were diluted in RIPA buffer (50 mM Tris-HCl pH 8.0, 150 mM NaCl, 1% NP-40, 0.5% sodium deoxycholate, 0.1% SDS, 2 mM EDTA) to a final volume of 15 µl. The diluted sample was denatured by heating at 95°C for 3 minutes before the addition of 3.17 µl of 7x cOmplete mini protease inhibitor mix and 4 µl of N-Glycosidase F (1 U/µl)). The reaction mix was incubated at 37°C for 1 h. The sample was then mixed with 7.40 µl of 4x RLB, heated at 95°C for 3 min and analyzed by SDS-PAGE and Western blotting. Zymographic assay for plasminogen activator was performed as previously described [27] with minor modifications. In brief, samples (50 µg) were electrophoresed on a 10% SDS-PAGE gel under non-reducing conditions. The gel was then washed twice with 2.5% Triton X-100 for 15 min, followed by two washes with PBS for 5 min. The electrophoretic gels were then layered on a substrate gel consisting of 1% low-melting point agarose, 2.5% non-fat milk, 40 µg/ml plasminogen (Roche, New Zealand) and 0.01% sodium azide. The zymogram was allowed to develop at 37°C for up to 120 h and images taken using a Fujifilm LAS-3000 with white-light illumination. Images were grey-scale inverted for presentation purposes.

### Quantitative Real-time Reverse-transcriptase Polymerase Chain Reaction (qRT-PCR)

Fresh hippocampi were collected from rats 61 days after AAV injection. These rats had been subjected to the open-field test, the novel object recognition test and the Morris water maze. Total RNA was extracted using TRIZOL Reagent (Invitrogen, CA, USA) and treated with DNase I (Invitrogen, CA, USA) to eliminate contamination by genomic DNA. cDNA synthesis was performed using the SuperScript III First-Strand Synthesis System (Invitrogen, CA, USA) and qRT-PCR was undertaken using the following TaqMan assays (Applied Biosystems, CA, USA): neuroserpin, forward primer: AGATCAGCATGATGCTGGTACTGT (100 nM), reverse primer: AGTTTGCCCATTCTTCGATCAG (300 nM), probe: 6FAM-CTCTGCTCAAACCAC (100 nM); tPA, forward primer: GGCCAAATGCCATCAAGCT (300 nM), reverse primer: CGTCTCGGTCTGGGTTTCTG (300 nM), probe: 6FAM-TGGGAATCACAATTACT (100 nM); PAI-1, Applied Biosystems Assays-on-Demand with Assay ID of Rn00561717_m1. qRT-PCR was performed in a 12 µl reaction mix containing: 20 mM Tris-HCl, pH 8.4, 50 mM KCl, 3 mM MgCl_2_, 200 µM dNTPs, 0.36 U of Platinum Taq DNA polymerase, 2.4 ng of cDNA, 1x ROX reference dye and the relevant primers and probes. Samples were initially heated to 94°C for 2 min to completely denature the templates and activate the enzyme. Cycling conditions were 94°C for 15 seconds and 60°C for 1 minute for 45 cycles using an Applied Biosystems 7900HT Fast Real-Time PCR System. Data analysis was carried out with the Sequence Detection Systems version 2.2.1 software (Applied Biosystems, CA, USA).

### Behavioural Testing

A total of 58 rats were used for the behavioural tests, split between two testing regimes (Figure S1B). Group size estimates were conducted using data based on previous similar AAV-based approaches to enhance learning and memory to give 80% power in detecting a medium effect size at an alpha level of 0.05 [16]. Behavioural tests were carried out three weeks after injection by an experimenter blinded to the treatments and in the dark phase of the light/dark cycle under dim lighting [24]. Each rat was handled for 5 min per day for five consecutive days prior to testing. Rats were randomly assigned to receive AAV-empty, AAV-hrGFP or AAV-FLAG-NS. The tests were carried out in an order to minimize the influence of performance of one test to the other and a recovery period of several days was provided between each test. The testing regime is outlined in Figure S1B. The first test for one set of animals investigated novel object recognition. In this test rats are initially required to interact with two objects. Any animals that did not fulfil this requirement of exploring both objects in the sample phase were excluded from further analysis. As this resulted in 3–4 animals from each group being excluded from the study and weakening the statistical power, this test was not included in the final data set.

#### Morris water maze

Morris water maze testing was carried out using procedures described previously [24] with minor modifications. The latency, path-length, distance and swim-speed to find the platform were recorded for each trial using Morris Watermaze 4.0 Software (Coulbourn Instruments, PA, USA). On day 1, rats were habituated to the apparatus using a visible platform placed at the centre of the pool. On day 2, each rat received non-spatial training to determine whether there was any motor function or motivational differences prior to testing. The visible platform was randomly located at the central position of one of four quadrants of the pool for each trial so that it was positioned in each quadrant once. The rat was put into water at the same fixed starting point for all four trials and allowed to search for the platform for 1 min. If successful in locating the visible platform, the rat was allowed to stay on the platform for 1 min; if not, it was directed to the platform by the experimenter where it was allowed to stay for 1 min. Each rat received four trials with a 1-min inter-trial interval. Acquisition training took place from day 3–7, with each rat given three trials per day with the submerged platform located at the centre of one quadrant throughout testing. For each trial, each rat was randomly placed into the pool in one of three starting points and allowed 1 min to locate the platform (if not located in this time, then the rat was guided to the platform by the experimenter). The rat was allowed to stay on the platform for 1 min before being returned to the holding cage for 1 min before the next trial. On day 8, each rat received a single probe trial. The platform was removed and rat allowed to swim for 1 min. The time spent in the quadrant previously containing the platform was recorded and served as a measure of memory retention. A spatial-cued test was carried out on day 9. Spatial cues were placed on a curtain and the visible platform was randomly located at the central position of one of four quadrants of the pool for each trial so that it was positioned in each quadrant once. The rat was put into water at a fixed starting point for all four trials (with an inter-trial interval of 1 min) and allowed to search for the platform for 1 min and allowed to stay on the platform for 1 min.

#### Contextual fear conditioning

Contextual fear conditioning was carried out as previously described [28] with slight modifications. Experiments were carried out in a small chamber (28 cm × 22 cm × 19 cm, L × W × H) with a transparent Plexiglas door to allow observation. The floor of the chamber consisted of 18 stainless steel rods (0.5 cm in diameter, spaced 1.2 cm apart), through which an electric foot shock could be delivered. Each rat was placed in the chamber for 170 s before the onset of an auditory tone which lasted for 10 s. The last 2 s of the auditory tone was paired with a mild electric shock (0.8 mA). The rat was allowed to stay in the chamber for an additional 120 s during which freezing behaviour (immediate response) was recorded, before the rat was returned to the home cage. Contextual fear memory was measured at 2 h and 24 h post-conditioning, by scoring freezing behaviour, defined as the absence of visible movement for 5 min except for respiration in the chamber in the absence of any auditory cue or foot shock.

#### Open field test

Rats were placed in a circular arena (190 cm diameter, 30 cm height). The floor of the arena was marked with lines that divided the open space into 4 concentric circles and into 43 segments of equal sizes. Rats were placed in the arena and allowed 5 min of free movement. Motor activity was quantified by counting the number of times the rat’s two front paws crossed a line separating 2 segments. The approach-avoidance conflict towards the open space was also assessed. The arena was divided into three zones: (1) the exploration zone in the centre consisting of 50% of the surface area, (2) the home zone which was the 15-cm wide area nearest to the side wall and (3) the intermediate zone which was the area between the exploration zone and the home zone. The percentage of time spent in each zone was recorded and was transformed into a zone preference index which allows comparison irrespective of the size of each zone [14]:
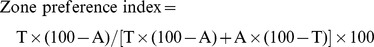
Where T = percentage of time spent in the zone and A = percentage of surface area occupied by the zone. An index of 0 represents complete avoidance of the zone and an index of 100 indicates complete preference for the zone.

#### Elevated T-maze

Assessment of innate and acquired anxiety was evaluated in the elevated T-maze as previously described [24]. The apparatus was cleaned with 70% ethanol between trials to remove any odour cues. On day 1, each rat was placed at one end of an enclosed arm, and the time taken for the rat to exit the arm (all four paws located outside the arm) was recorded (baseline avoidance latency). The animal was then returned to their home cage for 30 s before being placed on the enclosed arm. This procedure was repeated until the rat stayed in the enclosed arm continuously for 5 min without entering an open arm. The number of trials required for the rat to reach the criterion of staying in the enclosed arm for 5 min was recorded. The animal was then returned to the home cage for 30 s, before being placed at the end of an open arm, and the time taken to enter the enclosed arm recorded (escape latency). On day 4, avoidance and escape latencies were measured again. In brief, each rat was placed at the end of the enclosed arm and the latency to enter an open arm was recorded. The rat was then placed at the end of the open arm and the time taken to leave this arm was recorded.

#### Statistical analysis

Statistical analyses were performed with GraphPad Prism 4 (GraphPad Software Inc, CA, USA). One-way ANOVA and repeated measure ANOVA were used to analyse data. Significance was assigned at *p*<0.05 with Dunnett’s *post hoc* tests to assess significant differences between treatment groups.

## Results

### FLAG-tagged Neuroserpin Complexes with tPA and is Targeted to the Secretory Pathway

**Figure 1:**
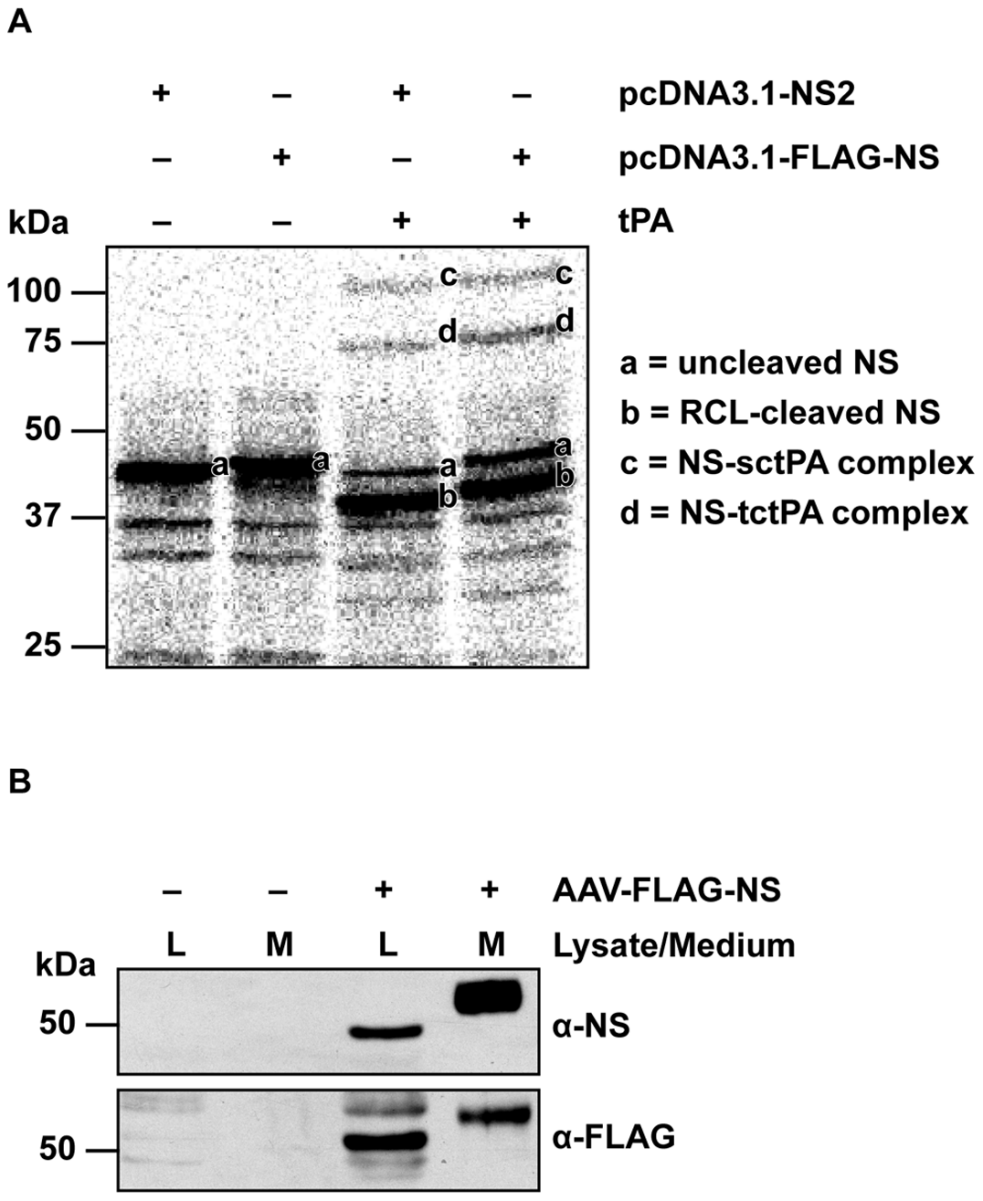
FLAG-tagged neuroserpin forms complexes with tPA and is secreted from transduced HEK293 cells. (A) Radiolabelled neuroserpins were incubated with or without tPA separated by a SDS-PAGE and imaged using a Fuji Phosphoimager (NS, neuroserpin; tPA, tissue plasminogen activator; RCL, reactive centre loop; sctPA, single-chain tPA; tctPA: two-chain tPA). (B) Lysate and medium samples collected from HEK293 cells that were either not transduced or transduced with AAV-FLAG-NS were separated by SDS-PAGE, followed by Western blotting with anti-NS and anti-FLAG antibodies.

To enable distinction between endogenous and exogenous neuroserpin following transduction of hippocampal neurons *in vivo*, a plasmid expressing a FLAG epitope-tagged-neuroserpin was engineered and characterized. The FLAG epitope was inserted after the 20-amino acid signal peptide to enable neuroserpin to be targeted to the secretory pathway. To verify that the insertion of the NH_2_-terminal FLAG epitope did not affect the inhibitory activity of neuroserpin we expressed ^35^S-labelled neuroserpin and FLAG-tagged neuroserpin *in vitro* and measured complexation with the target protease tPA (Figure 1A). The major radiolabelled band following *in vitro* transcription translation of wild type neuroserpin was ∼46 kDa**,** close to the predicted molecular weight of 46280 Da. As expected FLAG-tagged neuroserpin, which contained an extra 10 amino acids had an estimated molecular weight of ∼47 kDa. Incubation of radiolabelled neuroserpin with tPA resulted in several new radiolabelled bands consistent with the complex interaction known to occur between serpins and their target protease(s) [29], [30]. Neuroserpin formed covalent complexes with single and two chain forms of tPA. Dissociation of this complex resulted in the production of a reactive centre loop-cleaved carboxyl terminal-truncated form of neuroserpin. The same four bands were seen following incubation of FLAG-tagged neuroserpin with tPA confirming it was functionally active as a tPA inhibitor. The only difference was a small increase in the molecular weight of all the bands consistent with the incorporation of the FLAG sequence. To verify secretion FLAG-tagged neuroserpin HEK293 cells were transduced with recombinant AAV (Figure 1B). Immunoreactive neuroserpin was detected in both the cell lysate and medium using affinity-purified antibodies raised against the carboxyl terminus of neuroserpin and the FLAG epitope. With both antibodies immunoreactive neuroserpin in the medium had a higher molecular weight (∼53 kDa**)** than the cell lysate **(**∼49 kDa**)** consistent with posttranslational maturation of glycans in the secretory pathway [23], [31].

### Increased Hippocampal-localised Neuroserpin Transgene Expression Following Injection of AAV Vector Constructs into the Rat Dorsal Hippocampus

**Figure 2:**
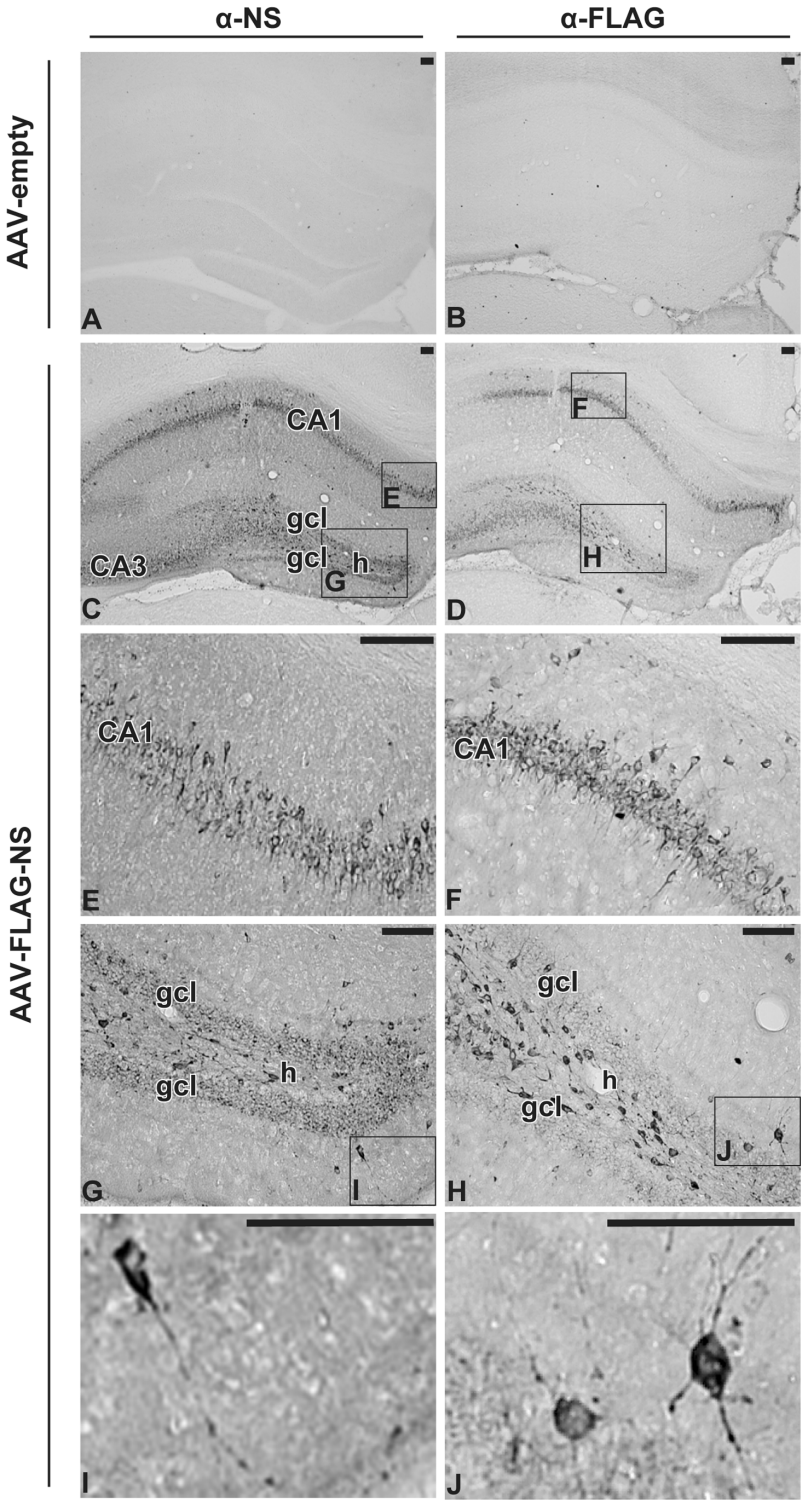
Expression of endogenous neuroserpin and FLAG-tagged-neuroserpin in the rat hippocampus. Immunohistochemical localization of neuroserpin and FLAG-tagged neuroserpin in rat brain sections near the AAV injection sites 21days after unilaterally injection with AAV-empty (A–B) or AAV-FLAG-NS (C–J) and detected using antibodies against neuroserpin (A, C, E, G, I) or FLAG (B, D, F, H, J). Representative brain sections from rats injected with AAV-empty (n = 2) or AAV-FLAG-NS (n = 2) are shown. CA1, pyramidal cell layers in CA1 subfield; CA3, pyramidal cell layers in CA3 subfield; gcl, dentate gyrus granule cell layer; h, hilus. Scale bar, 100 µm.

**Figure 3:**
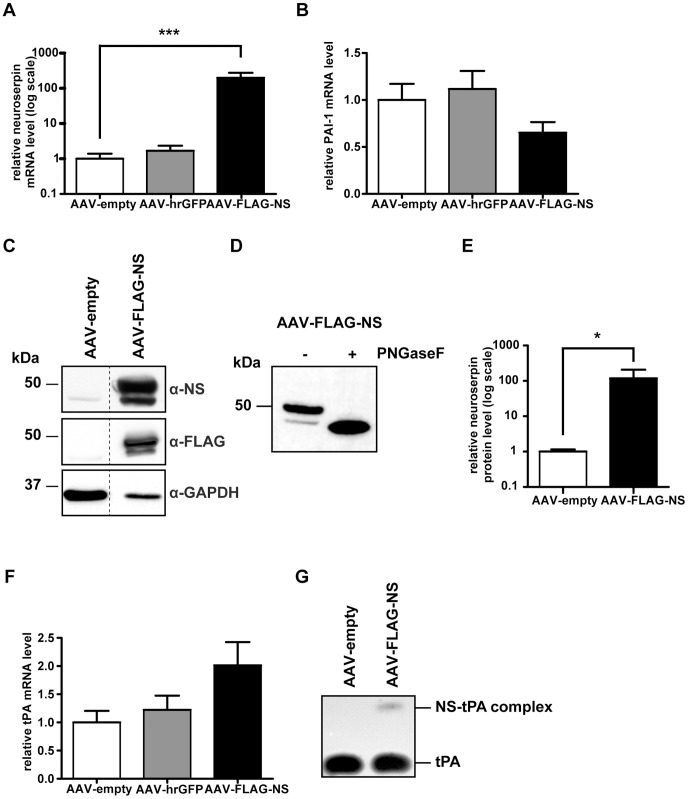
Increased neuroserpin expression and unchanged PAI-1 and tPA levels in AAV-FLAG-NS-transduced hippocampi. (A,B) qRT-PCR of neuroserpin (A) and PAI-1 (B) transcripts from hippocampi, 61 days after injection with AAV-empty (n = 3), AAV-hrGFP (n = 3) or AAV-FLAG-NS (n = 3). (C) Western blot analysis of endogenous and FLAG-tagged neuroserpin and glyceraldehyde-3-phosphate dehydrogenase (GAPDH) in hippocampal lysates (40 µg) from rats injected with AAV-empty (n = 3) or AAV-FLAG-NS (n = 3). (D) Western blot analysis of immunoreactive neuroserpin following N-linked deglycosylation of AAV-FLAG-NS hippocampal lysates (40 µg) with peptide-*N-*Glycosidase F (PNGaseF). (E) Quantitation of neuroserpin expression in panel C by measurement of the intensity of neuroserpin immunoreactive bands and normalization to GAPDH expression. (F) Quantitation of tPA transcripts from hippocampi injected with AAV-empty (n = 3), AAV-hrGFP (n = 3) or AAV-FLAG-NS (n = 3). (G) tPA enzymatic activity in the hippocampi lysates (50 µg) from rats injected with AAV-empty (n = 2) or AAV-FLAG-NS (n = 2). Lanes separated by dotted lines are from the same blot but with the lane order changed for presentation. All values are presented as the mean ± SEM. ***indicates *p*<0.001; *indicates *p*<0.05.

Neuroserpin transgene expression was investigated using IHC 21 days after AAV injection and by qRT-PCR and Western blotting of samples from animals that had been subjected to behavioural testing, 61 days after AAV injection. Immunohistochemical analysis of brain sections of rats injected unilaterally, in the dorsal hippocampus, with AAV-empty or AAV-FLAG-NS was performed using antibodies specific for neuroserpin and FLAG (Figure 2). The absence of endogenous neuroserpin immunoreactivity in the hippocampus of rats injected with recombinant AAV viral particles not expressing neuroserpin (Figure 2A) reflects the short development time used in these experiments to avoid overstaining of brain sections overexpressing neuroserpin. FLAG-immunoreactivity was also absent in sections from rats injected with AAV-empty, supporting the specificity of the anti-FLAG antibody (Figure 2B). Injection of AAV-FLAG-NS resulted in the expression of exogenous FLAG-tagged neuroserpin in hippocampal pyramidal cells in CA1-3, the dentate granule cells of the dentate gyrus and in the polymorphic region of the hilus (Figure 2C–H). FLAG-tagged neuroserpin expression was not only located in the cell body but also distributed to processes. (Figure 2I–J). Expression levels of neuroserpin, tPA and PAI-1, a serpin that is closely related to neuroserpin which also inhibits tPA [32], were also determined in hippocampi of rats that had been injected either with AAV-empty, AAV-hrGFP (acting as a control for overexpression of a protein mediated by AAV) or AAV-FLAG-NS. qRT-PCR showed a very large increase in the neuroserpin mRNA level in the rats injected with AAV-FLAG-NS, ∼200-fold relative to that of rats injected with AAV-empty (Figure 3A). There was no significant change in neuroserpin mRNA levels in rats injected with the AAV-hrGFP construct. There were also no significant differences in the levels of PAI-1 transcripts in hrGFP- and NS-expressing groups compared to the empty vector control (Figure 3B). Western blot analysis detected a faint neuroserpin-immunoreactive band with a molecular weight of ∼45 kDa in hippocampal lysates from rats injected with AAV-empty, indicating low basal expression of endogenous neuroserpin (Figure 3C). Hippocampal lysates from rats injected with AAV-FLAG-NS showed two intense neuroserpin-immunoreactive bands, with molecular weights of ∼45 kDa and ∼49 kDa. These two bands were also FLAG-immunoreactive, indicating they were exogenous forms of neuroserpin. To determine whether these bands represented differentially glycosylated forms of neuroserpin, AAV-FLAG-NS lysate was subjected to N-linked deglycosylation. Deglycosylation resulted in a single band of smaller size, supporting their identity as N-linked neuroserpin glycoforms (Figure 3D). Quantification of neuroserpin immunoreactivity (both bands combined) revealed that the AAV-FLAG-NS group had a ∼120-fold increase in the neuroserpin protein level when compared to the empty vector group (Figure 3E). We also analysed the mRNA levels and caseinolytic activity of tPA in hippocampal lysates. There was a ∼two-fold increase of tPA mRNA in samples collected from the neuroserpin group in comparison to the control (Figure 3F). However, ANOVA revealed that the *p* value was just below statistical significance (*p* = 0.076). There was no significant change in tPA mRNA levels in the hrGFP group. The effect of neuroserpin overexpression on tPA proteolytic activity was assessed by casein-plasminogen zymography. A single caseinolytic band consistent with the migration position of uncomplexed tPA (based on comparisons to the recombinant tPA control) was detected in the AAV-empty group (Figure 3G). This band was of similar intensity in the AAV-FLAG-NS treatment group. A second band of higher molecular weight was observed only in the AAV-FLAG-NS group. It migrated with an apparent molecular weight in the range of previously characterized complexes of neuroserpin with tPA reported in our earlier study [20] and by others [33]. The detection of this complex in AAV-FLAG-NS tissue lysates confirms that FLAG-tagged neuroserpin is able to complex with tPA *in vivo*, consistent with the *in vitro* biochemical data (Figure 1A).

### Postsynaptic Density Protein 95 (PSD-95) Levels were Reduced in the Hippocampus Rats Expressing FLAG-neuroserpin

**Figure 4:**
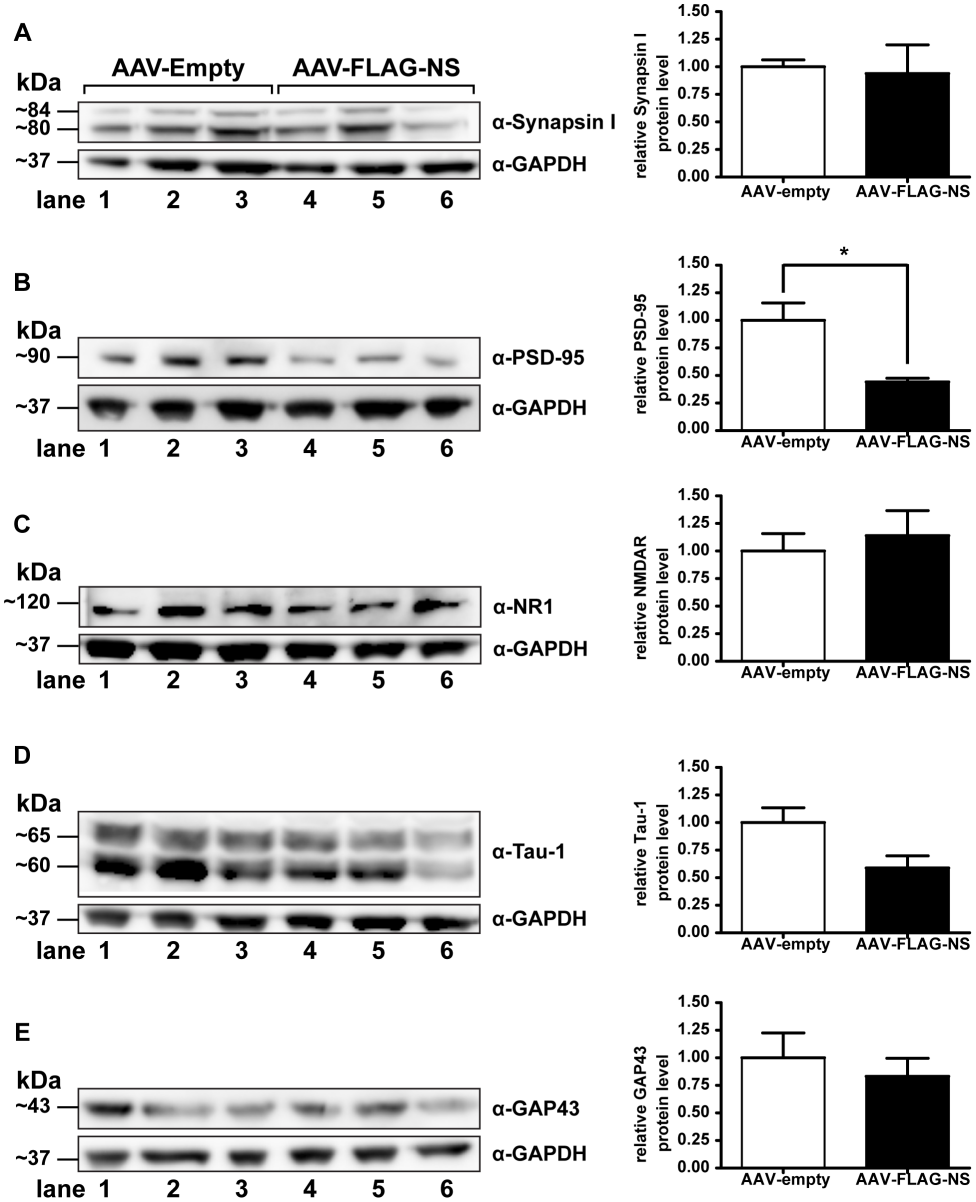
Expression of proteins associated with synaptic plasticity in the hippocampi of AAV-injected rats. Hippocampal lysates (35 µg) from rats injected with AAV-empty (n = 3) or AAV-FLAG-NS (n = 3) were analyzed by SDS-PAGE and Western blotting. Each lane represents protein expression from the same rat. Blots were probed with antibodies for synapsin I (A), PSD-95 (B), NR1 (C), Tau-1 (D) and GAP-43 (E) and immunoreactive bands normalized to GAPDH expression. Values are presented as the mean ± SEM. *indicates *p*<0.05.

The effect of neuroserpin overexpression on a number of proteins known to be involved in synaptic plasticity was investigated in hippocampal lysates prepared from rats injected either with AAV-empty or AAV-FLAG-NS that had previously undergone the open field test, novel object recognition test and the Morris water maze (Figure 4A–E). No significant difference in the levels of immunoreactive synapsin I, NR1 or GAP-43 was seen between rats injected with AAV-empty and rats injected with AAV-FLAG-NS. For Tau-1, levels decreased to 58.8% ±11.0% of the AAV-empty group. However, this decrease was largely the result of a marked decrease in neuroserpin expression in one animal and was not statistically significant. Levels of immunoreactive PSD-95 were significantly lower in the neuroserpin-treated group decreasing to 44.1% ±3.4% compared to the AAV-empty control group.

### Learning and Memory was not Affected by the Overexpression of Neuroserpin in the Hippocampus

Performance in a range of behavioural tests was measured on groups of rats injected bilaterally with AAV vectors. Two standard control vectors were used, a AAV empty vector containing the identical expression cassette as the AAV vector expressing neuroserpin but with no transgene and a AAV vector expressing enhanced green fluorescent protein GFP [16], [34]–[36]. The main behaviours of interest were learning and memory including spatial learning and memory, emotional memory and associative learning and memory. Prior to testing hippocampal-dependent behaviours, locomotor activity, exploratory behaviour and anxiety were assessed in the open field test [37] to ensure that there was no physical and motivational difference between groups that may influence interpretation of the tests. No significant differences were seen in the number of lines crossed in the open field test between the empty, hrGFP and neuroserpin groups suggesting there were no effects of neuroserpin overexpression on motor activity (data not shown).

**Figure 5:**
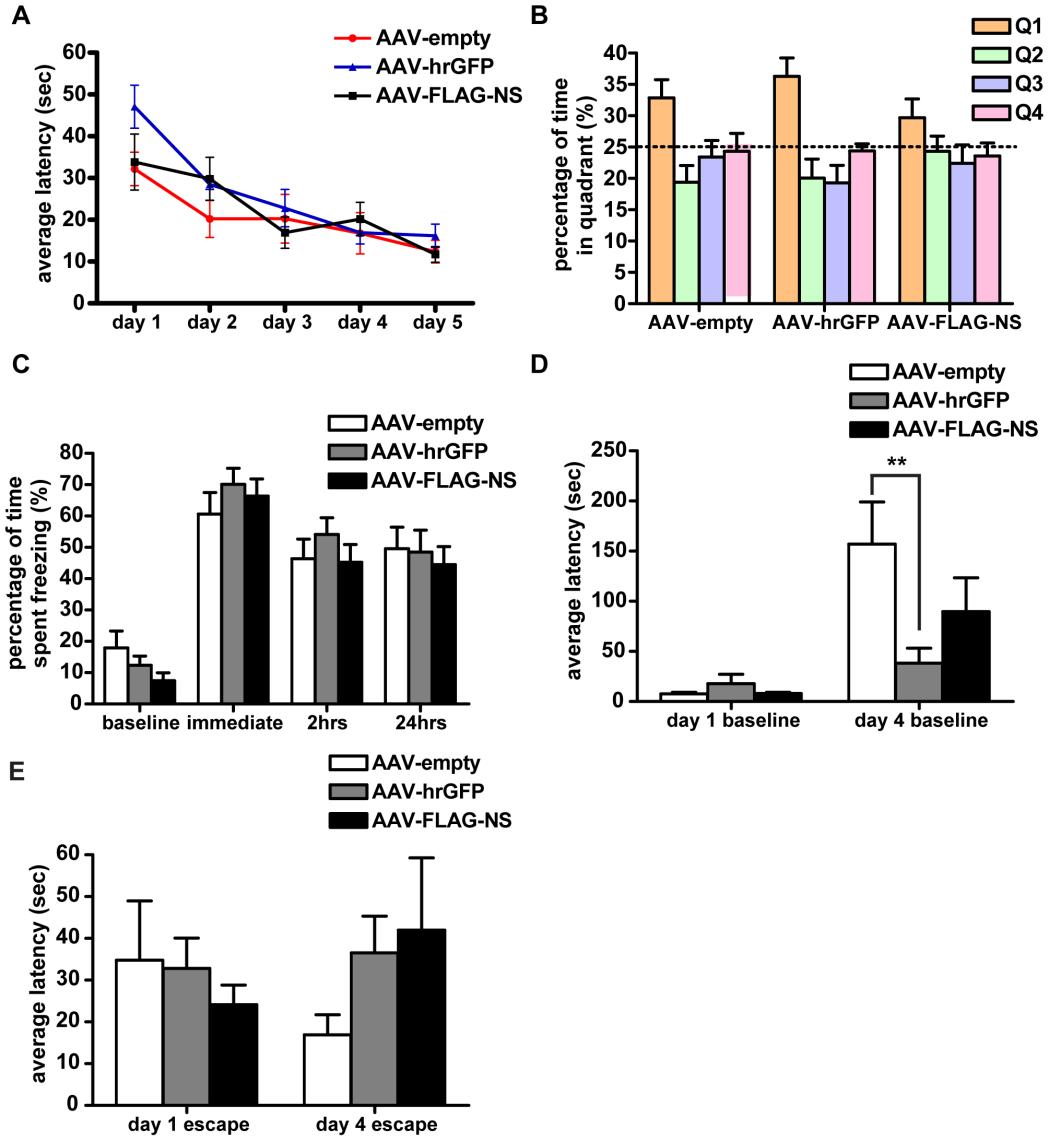
Learning and memory and anxiety and fear levels were not affected by neuroserpin overexpression. (A) All groups showed a decrease in swimming duration in the Morris water maze across the 5-day acquisition phase of spatial navigation but there was no significant difference between treatment groups (AAV-empty, n = 8; AAV-hrGFP, n = 7; AAV-FLAG-NS, n = 8) (F_8,80_ = 1.038, *p* = 0.4148). (B) In the Morris water maze there was no statistical significance in the time spent in Q1, the quadrant where the hidden platform was located, between the treatment groups (F_2,20_ = 1.222, *p* = 0.3156). (C) The contextual fear conditioning test showed no difference between treatment groups in the percentage of time the rats displaying freezing behaviour before, immediately after, 2 h and 24 h after the stimulus (AAV-empty, n = 11; AAV-hrGFP, n = 11; AAV-FLAG-NS, n = 13) (F_6,96_ = 1.218, *p* = 0.3039). (D) The elevated T-maze revealed no difference in innate and acquired fear between treatment groups (AAV-empty, n = 11; AAV-hrGFP, n = 11; AAV-FLAG-NS, n = 13). The average avoidance latency measured on day 1 and day 4 showed a significant treatment-by-inhibitory avoidance interaction (F_2,32_ = 0.3816, *p* = 0.0326). A significant difference was seen between the control and hrGFP group at day 4 (*t* = 3.491, *p* = 0.01) but there was no difference between control and neuroserpin group. (E) There was no significant difference in the average escape latency from the open arm to the enclosed arm at day 1 and day 4 between the treatment groups (F_2,32_ = 1.357, *p* = 0.2719). All measurements are presented as the mean ± SEM. **indicates *p*<0.01.

In the Morris water maze, rats are required to learn an optimal search strategy to find and recall the location of a hidden escape platform over 5 training blocks. For all the AAV-treated rats, the latency to locate the platform decreased over the 5 days of training (Figure 5A). Even though the empty vector-treated rats learned the location of the platform faster than the hrGFP and NS animals, reaching a minimum latency of ∼20 sec to locate the platform on day 2, performance of these empty-vector-treated rats was not improved beyond day 2. Moreover, the difference between treatment groups was not statistically significant. Swim speed remained constant over the 5 days (data not shown). The animals were tested in a probe trial after the last training trial to assess retention of spatial learning. Empty vector and hrGFP vector injected rats spent ∼33–37% of their time in the target quadrant, while NS vector-injected rats spent the least amount of time (∼30%) in this quadrant although there was no statistical difference between the groups (Figure 5B). These data indicated no difference between groups in memory retention. Associative learning and memory was measured by the contextual fear conditioning test. The percentage of time in which the rats displayed freezing in response to the stimulus increased dramatically (Figure 5C). This indicated that the rats remembered the aversive stimulus and showed an increased amount of fear. The treatment-by-time interaction was not significant, indicating freezing behaviour was not different between treatment groups and that overexpression of hrGFP or neuroserpin does not affect contextual fear conditioning.

### Overexpression of Neuroserpin did not Affect Anxiety and Fear Level in Rats

The open field test provides a measure of innate anxiety levels, as rats display a conflict between the tendency to explore a novel environment and the tendency to avoid an open area. Assessment of the preference for defined zones (home, intermediate and exploration zones) provides a means to determine their innate anxiety levels. All rats in each treatment group showed a strong preference to stay in the home zone close to the walls of the test arena, with the least amount of time spent in the exploratory zone in the centre of the field (Figure S2). However, there was no difference in zone preference between treatment groups, indicating that the treatments did not affect anxiety levels or locomotor activity. Innate and acquired anxiety was also assessed by performance in the elevated T-maze. A training-to-criterion procedure was adapted where rats were trained to stay in the enclosed arm continuously for 5 min as previous studies have shown that rats trained to a learning criterion show significant better retention and avoidance memory [38]. During avoidance training at day 1, there was no significant difference between treatment groups in terms of the latency to leave the closed arm or the number of trials required to reach the criterion of 5 min, indicating that overexpression of either hrGFP or neuroserpin did not affect the learning of conditioned fear. At day 4, the latency for the animal to move away from the enclosed arm was measured again and compared to that measured at day 1 (Figure 5D). The latency to leave the closed arm at day 4 was significantly higher than that at day 1, suggesting that the rats remembered the task and preferred to stayed in the closed arm. Interestingly, the treatment-by-inhibitory avoidance interaction was significant and suggested that there was a difference in the memory retention between the three treatment groups. There was a significant difference between the empty and hrGFP group at day 4 with the hrGFP having a shorter latency time, suggesting poorer memory in this group. However, no difference was detected between the empty and the neuroserpin treatment group. Unconditioned fear was also evaluated from the elevated T-maze by measuring the time required to escape from the open arm at day 1 and day 4 (Figure 5E). There was no difference in escape latency at day 1 and day 4. Moreover, the treatment-by-innate fear interaction was not significant, suggesting that the overexpression of either hrGFP or neuroserpin had no effect on innate anxiety.

## Discussion

In this study we have used a chimeric AAV1/2 vector to achieve localized overexpression of neuroserpin in the hippocampus of young adult rats and investigated the effect of neuroserpin overexpression on the expression of proteins linked to synaptic plasticity and hippocampal-dependent behaviours. To the best of our knowledge this is the first report to directly investigate a role for neuroserpin in hippocampal-dependent learning and memory in rats. Previous studies reporting neuroserpin expression in the hippocampus [2], [3], [5], regulation of neuroserpin expression [39] and secretion [40], [41] by neuronal activity, and the association of neuroserpin with activity-dependent plasticity of the primary visual cortex [12] led to suggestions that neuroserpin may play a role in hippocampal-dependent learning and memory [32]. However our results do not support such a role in the young adult rat. No changes were seen in behavioural tests targeting spatial and associative learning and memory. We also saw no changes in anxiety-like behaviour. The lack of effect on anxiety-related behaviours differs from a previous study which reported that transgenic mice overexpressing neuroserpin displayed neophobic behaviour with increased avoidance behaviour in the open field test and reduced investigation of the novel object during the novel object test [14]. While there were some small differences in the way the tests were performed it is more likely that the species difference along with differences in temporal and spatial expression of neuroserpin underlie the behavioural differences between the two studies. Our approach, restricting neuroserpin overexpression to the hippocampus, was designed to investigate a role for neuroserpin in hippocampal-dependent learning and memory in young adult rats. This contrasts with the transgenic mouse model where chicken neuroserpin overexpression was under the control of the Thy-1.2 promoter, which drives neuron-specific transcription starting at postnatal days 4–10 in virtually all regions of the brain [3], [33]. Transgene expression beginning at this time will not impact early brain development, but it could impact activity-dependent rearrangements of synaptic connections and other developmental events that occur during the late phase of nervous system development [42]. Increased neuroserpin expression in other brain regions such as the amygdala, a brain region implicated in fear and anxiety and in particular the unconditioned anxiety responses detected in the open field and elevated maze tests [43], [44] may account for the increased anxiety in these transgenic animals.

At the cellular level, we investigated the impact of neuroserpin overexpression on the expression and enzymatic activity of tPA. While there was a small increase in tPA mRNA levels there was no change in uncomplexed tPA enzymatic activity, as indicated by the unchanged intensity of the tPA band seen in casein zymography. However, there was clear evidence that FLAG-neuroserpin did interact with tPA *in vivo*, as shown by the presence of a higher molecular weight neuroserpin:tPA complex band in zymographs of hippocampal extracts from rats overexpressing FLAG-neuroserpin. A similar neuroserpin:tPA complex band has been reported in brain extracts from transgenic mice overexpressing chicken neuroserpin but not in control mice [33]. Detection of this complex is likely be due to an unusual feature of neuroserpin, which is the relative instability of the neuroserpin:tPA complex with a dissociation half-life of about 10 minutes, compared to weeks for most other serpin:protease complexes [45]–[47]. These results suggest that neuroserpin acts as a transient inhibitor of tPA. Therefore, it may not be entirely surprising that no changes were detected in uncomplexed tPA activity *in vivo* in the current study or in neuroserpin-knockout mice [14]. However, it remains unknown why uncomplexed tPA activity was reduced in transgenic mice overexpressing neuroserpin [33] but not the current study. One possible explanation is the different spatial expression patterns and levels of neuroserpin overexpression in the two studies. As neuroserpin overexpression was not limited to the hippocampus in the Cinelli study, it may be that the majority of the change in tPA levels in their brain homogenates was contributed by non-hippocampal areas. Under this scenario neuroserpin may still regulate tPA *in vivo*, but in a more fine-grained manner such as in specific compartments or across a shorter timescale.

We also investigated the expression levels of several proteins involved in neuronal plasticity. Of the proteins tested, PSD-95 showed a significant decrease in protein expression compared to the AAV-empty-injected control. PSD-95 is an abundant synaptic scaffolding protein that is enriched in the post-synaptic density (PSD) of excitatory glutamatergic synapses [48], [49]. In the nervous system, PSD-95 plays important roles in regulating the formation, function, and plasticity of excitatory synapses [49] and dendritic spines [50]–[54]. In the current study, it is not clear whether the effect of neuroserpin on PSD-95 levels occurs directly or indirectly. Neuroserpin may be directly altering the expression of PSD-95, leading to potential downstream effects on the signalling of AMPA-type glutamate receptor, synaptic plasticity or dendritic spine morphology. Alternatively, neuroserpin may cause a decrease in the number or size of dendritic spines, which is then reflected as a decrease in the PSD-95 level. In either case, this result suggests that neuroserpin might regulate the excitatory synapses of the hippocampus. This finding is supported by previous research showing that neuroserpin overexpression in hippocampal neurons alters the size of dendritic spines leading to a shift from mushroom-type spines to thin spines [23], which should also be correlated with a decrease in PSD-95 levels. PSD-95 has been suggested to bridge the low density lipoprotein receptor-related protein 1(LRP1) C-terminus to the N-methyl-D-aspartate (NMDA) receptor following the binding of tPA to LRP1, leading to tPA-induced calcium influx and activation of the mitogen-activated protein kinase (MAPK) signalling pathway [55]. Neuroserpin has also been shown to bind to LRP1 either as a complex with tPA or in association with an unidentified co-factor [56]. Neuroserpin could therefore affect the recruitment of PSD-95 to LRP1, and alter NMDA receptor-mediate calcium influx and MAPK signalling. More research will be required to investigate whether this is linked to the findings in the present study.

Interestingly, there was a lack of any changes in levels of two other synaptic proteins, synapsin I and NR1, that might have been expected to be impacted by a change in the level of PSD-95. Synapsin I is present in most CNS synapses including both excitatory and inhibitory synapses [57], and therefore an effect on the excitatory synapses may be masked by lack of changes in other synapse types. Although NR1 is localized to excitatory synapses, a number of studies have shown that changes in PSD-95 levels correlate with changes in the levels of other NMDA receptor subunits (e.g. NR2A and NR2B) but not NR1 levels [58]–[60]. This may be due to the presence of a large intracellular pool of reserve NR1 subunits, with the number of synaptic NMDA receptors determined by the expression of other NR subunits rather than NR1 [61], [62].

In summary, this study investigated the effects of localized overexpression of neuroserpin in the hippocampus on learning and memory. No behavioural changes were detected in four different behavioural tests designed to study locomotor activity, spatial and associative learning and memory, and innate and acquired fear. There was no evidence of neuroserpin overexpression in the hippocampus causing changes in anxiety level, fear or hippocampus-dependent learning and memory in the young adult rat. Nevertheless, a decrease in PSD-95 levels was triggered by neuroserpin overexpression supporting an involvement of neuroserpin in synaptic plasticity. Neuroserpin overexpression also led to the appearance of a tPA:neuroserpin complex confirming that FLAG-neuroserpin is able to interact with tPA *in vivo*. However it had no effect on uncomplexed tPA activity suggesting it may act as a transient inhibitor of tPA. Furthermore we cannot exclude the possibility that neuroserpin acts through a non-inhibitory mechanism. Previous research has found that neuroserpin can regulate cell-cell and cell-matrix interactions through a mechanism that does not involve inhibition of tPA [63].

## Supporting Information

Figure S1 **Schematic diagram of AAV injection and the testing regime of AAV-injected rats.** (A), AAV was injected bilaterally into the dorsal hippocampus at the locations(s) indicated (coordinates from bregma: antero-posterior −3.8 mm, medial-lateral ±2.0 mm and dorsal-ventral −4.1 mm). Image is reproduced from [26]. (B), The timeline of surgery and behavioural test performed on rats injected with AAV vectors. A total of 58 rats were injected with AAV-empty (n = 19), AAV-hrGFP (n = 18) or AAV-FLAG-NS (n = 21), and tested in the open field test. A subset of these AAV-injected rats (AAV-empty, n = 8; AAV-hrGFP, n = 7; AAV-FLAG-NS, n = 8) was tested in the object recognition test and the Morris water maze. The remaining AAV-injected rats (AAV-empty, n = 11; AAV-hrGFP, n = 11; AAV-FLAG-NS, n = 13) were tested in the elevated T-maze and the contextual fear conditioning test.
(TIF)

Figure S2 **Anxiety and fear levels were not affected by neuroserpin overexpression.** During the open field test (A), all the rats (n = 58) showed a strong preference to stay in the home zone while the exploration zone was the least preferred zone (F_2,55_ = 1407, *p* = 0.001). However, there was no significant difference in the zone preference between the three treatment groups (F_4,55_ = 0.7064, *p* = 0.5892).
(TIF)
