# Supplementary figures and images for: AAV-Mediated Overexpression of Neuroserpin in the Hippocampus Decreases PSD-95 Expression but Does Not Affect Hippocampal-Dependent Learning and Memory
Source: PLoS One. 2014 Mar 7;9(3):e91050. doi: 10.1371/journal.pone.0091050 (PMC3946662; doi:10.1371/journal.pone.0091050)

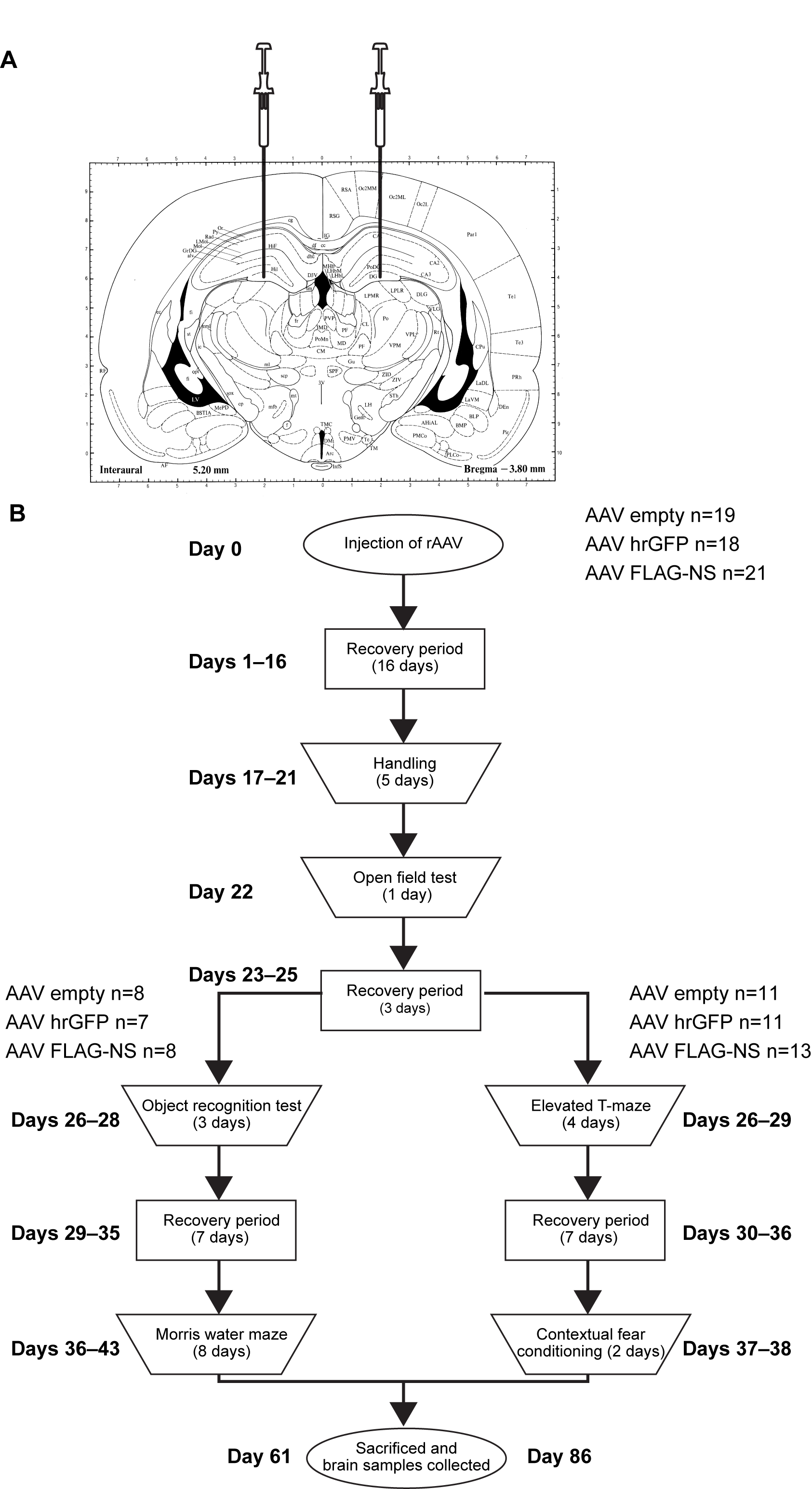

Supplement: Figure S1 — Schematic diagram of AAV injection and the testing regime of AAV-injected rats. (A), AAV was injected bilaterally into the dorsal hippocampus at the locations(s) indicated (coordinates from bregma: antero-posterior −3.8 mm, medial-lateral ±2.0 mm and dorsal-ventral −4.1 mm). Image is reproduced from [26]. (B), The timeline of surgery and behavioural test performed on rats injected with AAV vectors. A total of 58 rats were injected with AAV-empty (n = 19), AAV-hrGFP (n = 18) or AAV-FLAG-NS (n = 21), and tested in the open field test. A subset of these AAV-injected rats (AAV-empty, n = 8; AAV-hrGFP, n = 7; AAV-FLAG-NS, n = 8) was tested in the object recognition test and the Morris water maze. The remaining AAV-injected rats (AAV-empty, n = 11; AAV-hrGFP, n = 11; AAV-FLAG-NS, n = 13) were tested in the elevated T-maze and the contextual fear conditioning test. (TIF) [file pone.0091050.s001.tif]

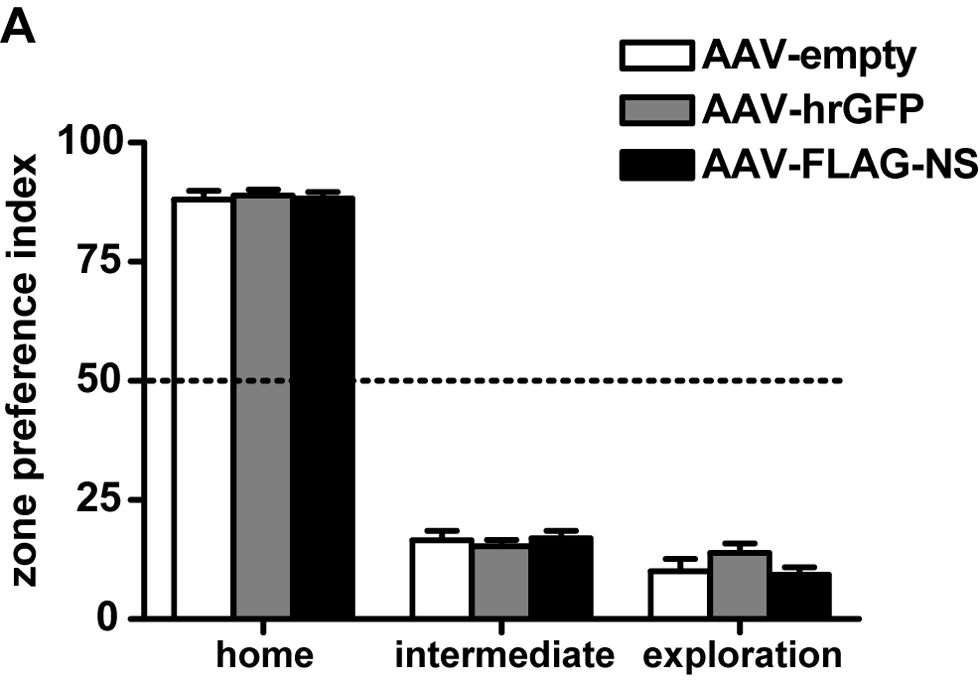

Supplement: Figure S2 — Anxiety and fear levels were not affected by neuroserpin overexpression. During the open field test (A), all the rats (n = 58) showed a strong preference to stay in the home zone while the exploration zone was the least preferred zone (F2,55 = 1407, p = 0.001). However, there was no significant difference in the zone preference between the three treatment groups (F4,55 = 0.7064, p = 0.5892). (TIF) [file pone.0091050.s002.tif]
